# Pien Tze Huang inhibits hypoxia-induced epithelial-mesenchymal transition in human colon carcinoma cells through suppression of the HIF-1 pathway

**DOI:** 10.3892/etm.2014.1549

**Published:** 2014-02-17

**Authors:** HONGWEI CHEN, ALING SHEN, YUCHEN ZHANG, YOUQIN CHEN, JIUMAO LIN, WEI LIN, THOMAS SFERRA, JUN PENG

**Affiliations:** 1 Academy of Integrative Medicine, Fujian University of Traditional Chinese Medicine, Fuzhou, Fujian 350122, P.R. China; 2 Fujian Key Laboratory of Integrative Medicine on Geriatrics, Fujian University of Traditional Chinese Medicine, Fuzhou, Fujian 350122, P.R. China; 3 Rainbow Babies and Children’s Hospital, Case Western Reserve University School of Medicine, Cleveland, OH 44106, USA; 4 Postdoctoral Workstation, Zhangzhou Pien Tze Huang Pharmaceutical Co., Ltd., Zhangzhou, Fujian 363000, P.R. China

**Keywords:** Pien Tze Huang, traditional Chinese medicine, colon cancer, hypoxia, epithelial-mesenchymal transition, HIF-1 pathway

## Abstract

Hypoxia-induced activation of the hypoxia-inducible factor 1 (HIF-1) signaling pathway is frequently observed in solid tumors and is strongly associated with numerous pathophysiological processes, including the induction of epithelial-mesenchymal transition (EMT), which result in cancer progression and metastasis. Thus, inhibiting EMT through the suppression of the HIF-1 pathway may be a promising strategy for anticancer chemotherapy. Pien Tze Huang (PZH), a well-established traditional Chinese medicine has been prescribed for >450 years and has been used for centuries to clinically treat various types of human cancer. We previously reported that PZH suppresses multiple intracellular signaling pathways and thereby promotes the apoptosis of cancer cells and the inhibition of cell proliferation and tumor angiogenesis. In the present study, to further explore the mechanisms underlying the antitumor action of PZH, HCT-8 human colon carcinoma cells were cultured under hypoxic conditions and the effect of PZH on hypoxia-induced EMT was assessed. Hypoxia was found to induce EMT-associated morphological changes in HCT-8 cells, including loss of cell adhesion and the development of spindle-shaped fibroblastoid-like morphology. In addition, hypoxia was observed to reduce the expression of the epithelial marker E-cadherin, but increase that of the mesenchymal marker N-cadherin. In addition, hypoxia significantly enhanced HCT-8 cell migration and invasion and induced the activation of the HIF-1 pathway. However, treatment of the HCT-8 cells with PZH significantly inhibited the hypoxia-mediated EMT and HIF-1 signaling. These findings suggest that PZH inhibits hypoxia-induced cancer EMT through the suppression of the HIF-1 pathway, which may be one of the molecular mechanisms by which PZH exerts its antitumor activity.

## Introduction

Hypoxia is a common characteristic of all rapidly growing solid tumors (1). Tumor hypoxia is caused by a number of factors, including inadequate blood supply due to abnormal tumor microvasculature, increased diffusion distances from the blood vessels to the tumor tissues and a reduced capacity of the blood to carry oxygen due to anemia (2). Intracellular hypoxic responses are highly regulated by hypoxia-inducible factors (HIFs), which are transcription factors with critical roles in the development and progression of cancer (3–5). HIFs belong to a family of basic helix-loop-helix-containing proteins (6). The prototypic member of this family is HIF-1, which is a heterodimer consisting of an oxygen-regulated α subunit and a constitutively expressed β subunit (7–9). While HIF-1β is constitutively expressed in cells, HIF-1α protein expression is dependent on intracellular oxygen concentration. Under normoxia, HIF-1α protein is continuously expressed, but rapidly degraded, as it is hydroxylated by prolyl hydroxylases (PHDs) at proline residues within the oxygen-dependent degradation domain, which in turn mediates its interaction with the von Hippel-Lindau (pVHL) tumor suppressor protein, eventually leading to HIF-1α ubiquitination and degradation through a VHL-dependent ubiquitin-proteasome pathway (10–12). However, under hypoxic conditions, the O_2_-dependent PHDs are inhibited, thus the interaction between HIF-1α and pVHL is prevented. Consequently, HIF-1α ubiquitination/degradation is inhibited, resulting in an increase in HIF-1α protein expression (6,13). The stabilized HIF-1α subunit then translocates to the nucleus where it heterodimerizes with the HIF-1β subunit (14) and subsequently regulates the expression of numerous important genes involved in the regulation of various biological processes (15). HIF-1α overexpression is commonly found in numerous types of human cancer and is often associated with tumor progression and poor prognosis (16,17). One of the mechanisms by which HIF-1 promotes cancer progression is through the induction of epithelial-mesenchymal transition (EMT), a process in which epithelial cells lose cell-cell adhesion and cell polarity, and acquire properties of mesenchymal cells (18–21). Through the process of EMT, carcinoma cells undergo migration and invasion, leading to cancer progression and metastasis (22). Thus, hypoxia-induced EMT may be a promising target for anticancer chemotherapy.

Due to the drug resistance and adverse side-effects associated with the majority of currently used cancer chemotherapies, natural products have gained great interest as they have comparatively few side-effects and have been used clinically to treat a variety of diseases, including cancer (23,24). Traditional Chinese medicines (TCMs) are complex combinations of various natural products, each of which contain numerous chemical compounds. Thus, TCMs are considered to be multi-component and multi-target agents that exert their therapeutic activities in a holistic way. Pien Tze Huang (PZH) is a well-established TCM that was first prescribed >450 years ago in the Ming Dynasty (25). PZH has been used in China and Southeast Asia for centuries as a remedy for various types of human cancer. We recently demonstrated that PZH suppresses multiple colorectal cancer-associated signaling pathways, leading to the promotion of cancer cell apoptosis and the inhibition of cell proliferation and tumor angiogenesis (26–31). In the present study, to further elucidate the mechanism underlying the antitumor activity of PZH, the effect of PZH on EMT under hypoxia was investigated in a human colon carcinoma cell line.

## Materials and methods

### Materials and reagents

Roswell Park Memorial Institute (RPMI)-1640 medium, fetal bovine serum (FBS), penicillin-streptomycin and TRIzol^®^ reagent, were purchased from Invitrogen Life Technologies (Carlsbad, CA, USA). HIF-1α, twist basic helix-loop-helix transcription factor (TWIST1), E-cadherin, N-cadherin and β-actin antibodies, as well as horseradish peroxidase (HRP)-conjugated secondary antibodies were purchased from Cell Signaling Technology Inc. (Danvers, MA, USA). Transwell^®^ chambers were obtained from Corning Life Sciences (Tewksbury, MA, USA). Matrigel™ was purchased from BD Biosciences (San Jose, CA, USA). SuperScript^®^ II reverse transcriptase was obtained from Promega Corporation (Madison, WI, USA). The Anoxomat™ Mark II hypoxic cell-culturing system was purchased from Mart Microbiology B.V. (Drachten, The Netherlands). Unless stated otherwise, all other chemicals were obtained from Sigma-Aldrich (St. Louis, MO, USA).

### Preparation of PZH

PZH was obtained from and authenticated by the sole manufacturer Zhangzhou Pien Tze Huang Pharmaceutical Co., Ltd., (Zhangzhou, China; Chinese FDA approval no. Z35020242). PZH stock solution was prepared prior to use by dissolving the PZH powder in phosphate-buffered saline to a concentration of 20 mg/ml. The working solutions of PZH were prepared by diluting the stock solution in the culture medium.

### Cell Culture

HCT-8 human colon carcinoma cells were obtained from Nanjing KeyGen Biotech. Co. Ltd. (Nanjing, China). Cells were cultured in RPMI-1640 containing 10% (v/v) FBS, 100 U/ml penicillin and 100 μg/ml streptomycin in a 37°C humidified incubator with 5% CO_2_. To induce cell hypoxia, cells were cultured in a multi-gas Anoxomat Mark II incubator, with 5% CO_2_ and 0.1% O_2_ balanced with N_2_.

### Observation of morphological changes

HCT-8 cells were seeded onto six-well plates at a density of 5×10^5^ cells/well in 2 ml medium. Cells were cultured under normoxia or hypoxia (0.1% O_2_), with or without treatment with various concentrations of PZH for 24 h. Cell morphology was observed using a Leica phase-contrast microscope (Leica Microsystems Ltd., Wetzlar, Germany). Images were captured at a magnification of ×400.

### Cell migration and invasion assays

Migration assays were performed using Transwell cell culture chambers with 8-μm pore filters (Corning Life Sciences). Following treatment with various concentrations of PZH under normoxia or hypoxia for 6 h, HCT-8 cells were trypsinized and resuspended in serum-free RPMI-1640. A total of 5×10^4^ cells in 200 μl serum-free RPMI-1640 were plated in the upper chambers. RPMI-1640 media containing 10% (v/v) FBS was used in the lower chambers as a chemoattractant. Cells were allowed to migrate for 12 h under normoxia, following which the non-migrating cells were removed from the upper surface of the Transwell membrane in each Transwell using a cotton swab. Membranes were then stained with crystal violet. For quantification, the average number of migrating cells per field was assessed by counting three random fields under a Leica phase-contrast microscope (Leica, Microsystems Ltd.) at a magnification of ×200. For the cell invasion assays, the procedure was the same as that used for the migration assay; however, the upper chambers were coated with 100 μl/well 0.2 mg/ml Matrigel Matrix (BD Biosciences) and cell invasion was allowed to progress for 24 h in normoxia.

### Reverse transcription (RT)-PCR analysis

Total RNA was isolated using TRIzol reagent. Oligo(dT)-primed RNA (1 μg) was reverse-transcribed using SuperScript II reverse transcriptase to generate cDNA according to the manufacturer’s instructions. cDNA was used to determine the quantity of HIF-1α, TWIST1, E-cadherin and N-cadherin mRNA using RT-PCR analysis with Taq DNA polymerase (Fermentas, Burlington, ON, Canada). GAPDH was used as an internal control.

### Western blot analysis

HCT-8 cells were seeded into 25 cm^2^ flasks at a density of 1.5×10^6^ cells/flask in 5 ml medium. Cells were cultured under normoxia or hypoxia (0.1% O_2_), with or without treatment with various concentrations of PZH for 24 h. Cells were then were lysed with mammalian cell lysis buffer containing protease and phosphatase inhibitor cocktails. Total protein concentrations were determined using a BCA protein assay. Equal quantities of total protein were resolved using 12% SDS-PAGE and electroblotted onto polyvinylidene fluoride membranes. Membranes were blocked using 5% skimmed milk and probed overnight at 4°C with primary antibodies against N-cadherin, E-cadherin, HIF-1α, TWIST1 and β-actin diluted 1:1,000. Membranes were then probed with the appropriate HRP-conjugated secondary antibodies and the immunoreactive bands were visualized using an enhanced chemiluminescence method (Bio-Rad, Hercules, CA, USA).

### Statistical Analysis

All data are presented as the mean ± standard deviation of three independent experiments and were analyzed using SPSS version 18.0 for Windows (SPSS, Inc., Chicago, IL, USA). Statistical data analyses were performed using the Student’s t-test and analysis of variance. P<0.05 was considered to indicate a statistically significant difference.

## Results and Discussion

### PZH inhibits hypoxia-induced EMT in HCT-8 human colon carcinoma cells

**Figure 1:**
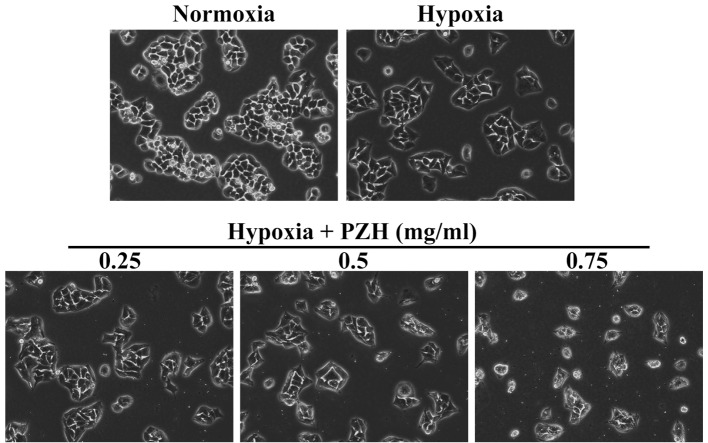
Effect of PZH on hypoxia-induced morphological changes in HCT-8 cells. HCT-8 cells were treated with the indicated concentrations of PZH for 24 h under normoxia or hypoxia (0.1% O_2_). Cell morphology was observed using phase-contrast microscopy. Images were captured at a magnification of ×400. Images are representative of three independent experiments. PZH, Pien Tze Huang.

**Figure 2:**
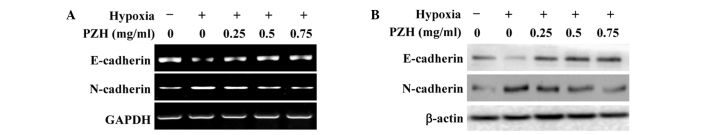
Effect of PZH on E-cadherin and N-cadherin expression in HCT-8 cells under hypoxia. HCT-8 cells were treated with the indicated concentrations of PZH for 6 h under normoxia or hypoxia. (A) mRNA and (B) protein expression levels of E-cadherin and N-cadherin were determined using RT-PCR and western blot analyses. GAPDH and β-actin were used as internal controls for RT-PCR and western blot analysis, respectively. Images are representative of three independent experiments. PZH, Pien Tze Huang; RT-PCR, reverse transcription-polymerase chain reaction.

Hypoxia is a common microenvironment for pathophysiological progresses, including tumor progression and metastasis (1,2). Metastasis is a complex process that involves the spread of malignant tumor cells from the primary tumor site to a secondary organ. This distant organ colonization is primarily initiated through EMT. Epithelial and mesenchymal cells are different in phenotype and function. Epithelial cells have an apical-basal polarity, express high levels of epithelial markers, including E-cadherin, and form epithelial adherent junctions. By contrast, mesenchymal cells lack cell polarity, overexpress mesenchymal markers, including N-cadherin and vimentin, and exhibit a spindle-like morphology (18–22). In the present study, in order to enable the effect of PZH on cancer EMT to be assessed, the morphological changes in HCT-8 cells under hypoxia were investigated. As shown in Fig. 1, under hypoxia HCT-8 cells exhibit greater isolation than under normoxia and a more spindle-shaped fibroblastoid-like morphology, which are typical characteristics associated with EMT. However, these hypoxia-induced EMT-associated morphological changes were observed to be inhibited by PZH treatment. To further verify these results, the expression of several critical genes that are involved in the regulation of EMT was investigated. As shown in Fig. 2, hypoxia was found to significantly reduce the expression of epithelial cell-specific E-cadherin, and increase that of the mesenchymal marker N-cadherin. However, the hypoxia-induced alterations in the expression of EMT-regulatory genes were attenuated by PZH treatment in the HCT-8 cells.

### PZH inhibits the hypoxia-enhanced migration and invasion of HCT-8 cells

**Figure 3:**
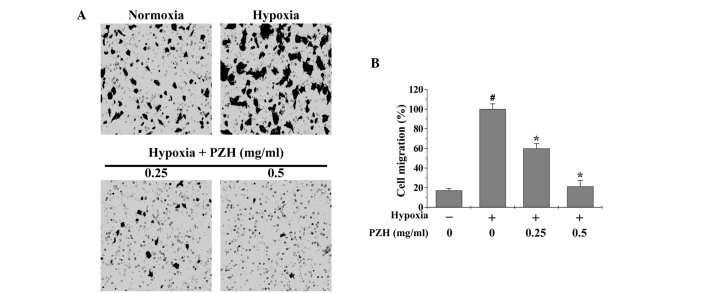
Effect of PZH on HCT-8 cell migration under hypoxia. HCT-8 cells were treated with the indicated concentrations of PZH for 6 h under normoxia or hypoxia. (A) Cell migration was determined using Transwell^®^ cell culture chambers. Cells were fixed and stained with crystal violet and images were captured at a magnification of ×200. (B) Average number of migrated cells from three randomly selected fields. Data were normalized to the migration of HCT-8 cells under hypoxia, but without PZH treatment. Data are presented as the mean ± standard deviation from three independent experiments. ^#^P<0.05 vs. normoxia; ^*^P<0.05 vs. hypoxia without PZH treatment. PZH, Pien Tze Huang.

**Figure 4:**
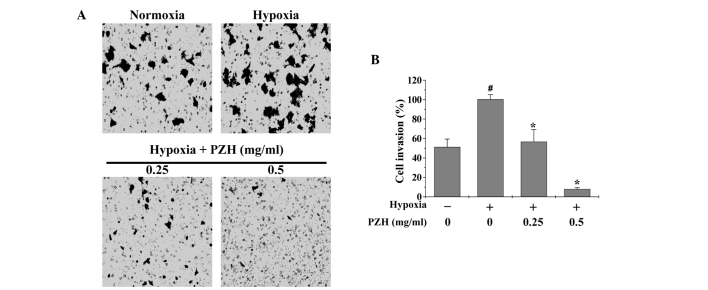
Effect of PZH on HCT-8 cell invasion under hypoxia. HCT-8 cells were treated with the indicated concentrations of PZH for 6 h under normoxia or hypoxia. (A) Cell invasion was determined using Transwell^®^ cell culture chambers with membranes coated with Matrigel™. Cells were fixed and stained with crystal violet and images were captured at a magnification of ×200. (B) Average number of migrated cells from three randomly selected fields. Data were nornalized to the invasion of HCT-8 cells under hypoxia, but without PZH treatment. Data are presented as the mean ± standard deviation from three independent experiments. ^#^P<0.05 vs. normoxia; ^*^P<0.05, vs. hypoxia without PZH treatment. PZH, Pien Tze Huang.

EMT promotes cancer cell metastasis; therefore, Transwell assays were performed in order to analyze the effect of PZH on the migration and invasion of HCT-8 cells under hypoxia. As shown in Figs. 3 and 4, hypoxia was observed to increase HCT-8 cell migration and invasion by 5.8- and 1.9-fold, respectively, compared with that of the cells cultured under normoxia (both P<0.05). However, treatment with 0.25–0.5 mg/ml PZH was observed to significantly decrease the cell migration and invasion rates by 40.1–78.7% and 43.3–92.1% (P<0.05), respectively, suggesting that PZH concentration-dependently inhibits the hypoxia-induced metastasis of colon cancer cells.

### PZH inhibits hypoxia-induced activation of the HIF-1α pathway in HCT-8 cells

**Figure 5:**
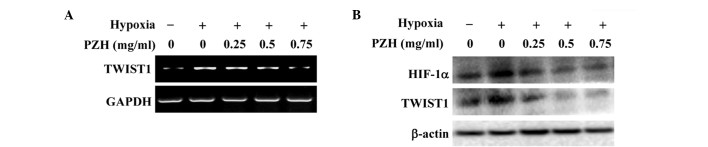
Effect of PZH on hypoxia-induced activation of the HIF-1 pathway in HCT-8 cells. HCT-8 cells were treated with the indicated concentrations of PZH for 6 h under normoxia or hypoxia. (A) mRNA and (B) protein expression of HIF-1α and TWIST1 were determined using RT-PCR and western blot analyses, respectively. GAPDH and β-actin were used as internal controls for RT-PCR and western blot analysis, respectively. Images are representatives of three independent experiments. PZH, Pien Tze Huang; HIF, hypoxia-inducible factor; TWIST1, twist basic helix-loop-helix transcription factor 1.

The intracellular response to hypoxia is primarily controlled by HIF-1, which consists of an oxygen-regulated α subunit and a constitutively expressed β subunit (3–5). It has been shown that the hypoxia-induced stabilization of HIF-1α is strongly associated with EMT (18–22). The transcription factor TWIST is one of the essential factors mediating EMT and cancer metastasis and it is highly regulated by HIF-1. Activation of TWIST represses the expression of epithelial markers, but upregulates the expression of mesenchymal markers (9,10). To further investigate the mechanism underlying the inhibitory activity of PZH against EMT, the effect of PZH on the activation of the HIF-1 pathway was investigated. As shown in Fig. 5, hypoxia was observed to significantly increase the mRNA and protein expression levels of HIF-1α and TWIST1; these increases were inhibited by PZH treatment in a concentration-dependent manner.

In conclusion, to the best of our knowledge, the present study has provided the first evidence that PZH is capable of inhibiting hypoxia-induced EMT in cancer cells through suppressing the activation of the HIF-1 pathway. This may be one of the molecular mechanisms underlying the antitumor activity of PZH.
