# [Corrigendum] Pien Tze Huang inhibits hypoxia‑induced epithelial‑mesenchymal transition in human colon carcinoma cells through suppression of the HIF‑1 pathway

**DOI:** 10.3892/etm.2024.12458

**Published:** 2024-02-28

**Authors:** Hongwei Chen, Aling Shen, Yuchen Zhang, Youqin Chen, Jiumao Lin, Wei Lin, Thomas Sferra, Jun Peng

Exp Ther Med 7:1237–1242, 2014; DOI: 10.3892/etm.2014.1549

Following the publication of the above article, the authors have realized that they inadvertently uploaded an incorrect figure for Fig. 3A (cell migration assay) as it appeared on p. 1240 for the ‘Hypoxia + PZH (mg/ml)/0.25’ data panel, as this panel was found to contain an overlap of data with the ‘Hypoxia + PZH (mg/ml)/0.25’ data panel in Fig. 4A (cell invasion assay), and requested that this error be rectified through publishing a corrigendum. The Editor of *Experimental and Therapeutic Medicine* has agreed to their request, and the corrected version of Fig. 3, now showing the correct data for the Hypoxia + PZH experiments (i.e., the lower panels), is shown below. Note that this error did not affect the overall conclusions reported in the paper. All the authors agree with the publication of this corrigendum, and are grateful to the Editor for granting them the opportunity to publish this. Furthermore, they also apologize to the readership for any inconvenience caused.

## Figures and Tables

**Figure 3:**
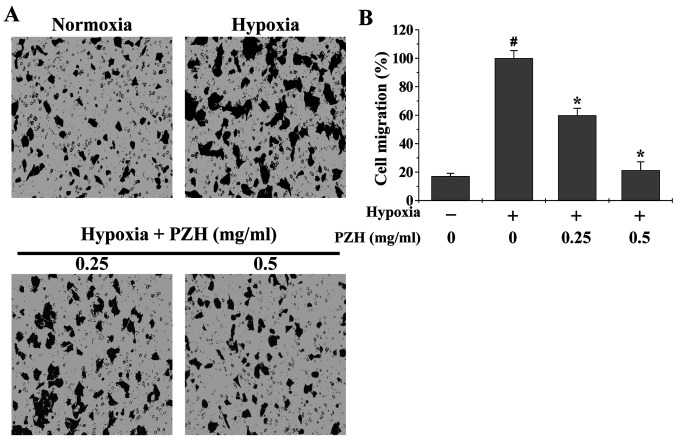
Effect of PZH on HCT-8 cell migration under hypoxia. HCT-8 cells were treated with the indicated concentrations of PZH for 6 h under normoxia or hypoxia. (A) Cell migration was determined using Transwell^®^ cell culture chambers. Cells were fixed and stained with crystal violet and images were captured at a magnification of ×200. (B) Average number of migrated cells from three randomly selected fields. Data were normalized to the migration of HCT-8 cells under hypoxia, but without PZH treatment. Data are presented as the mean ± standard deviation from three independent experiments. ^#^P<0.05 vs. normoxia; ^*^P<0.05 vs. hypoxia without PZH treatment. PZH, Pien Tze Huang.

